# Colorimetric Focus-Forming Assay with Automated Focus Counting by Image Analysis for Quantification of Infectious Hepatitis C Virions

**DOI:** 10.1371/journal.pone.0043960

**Published:** 2012-08-24

**Authors:** Wonseok Kang, Eui-Cheol Shin

**Affiliations:** Laboratory of Immunology and Infectious Diseases, Graduate School of Medical Science and Engineering, KAIST, Daejeon, Republic of Korea; Rosalind Franklin University of Medicine and Science, United States of America

## Abstract

Hepatitis C virus (HCV) infection is the leading cause of liver transplantation in Western countries. Studies of HCV infection using cell culture-produced HCV (HCVcc) *in vitro* systems require quantification of infectious HCV virions, which has conventionally been performed by immunofluorescence-based focus-forming assay with manual foci counting; however, this is a laborious and time-consuming procedure with potentially biased results. In the present study, we established and optimized a method for convenient and objective quantification of HCV virions by colorimetric focus-forming assay with automated focus counting by image analysis. In testing different enzymes and chromogenic substrates, we obtained superior foci development using alkaline phosphatase-conjugated secondary antibody with BCIP/NBT chromogenic substrate. We additionally found that type I collagen coating minimized cell detachment during vigorous washing of the assay plate. After the colorimetric focus-forming assay, the foci number was determined using an ELISpot reader and image analysis software. The foci number and the calculated viral titer determined by this method strongly correlated with those determined by immunofluorescence-based focus-forming assay and manual foci counting. These results indicate that colorimetric focus-forming assay with automated focus counting by image analysis is applicable as a more-efficient and objective method for quantification of infectious HCV virions.

## Introduction

Hepatitis C virus (HCV) is an RNA virus of the genus *Hepacivirus* in the family *Flaviviridae*, and it infects 170 million people worldwide [1]. HCV tends to develop chronic persistent infection and chronic hepatitis that often progresses to liver cirrhosis and hepatocellular carcinoma [2]. In Western countries, HCV infection is the leading cause of liver transplantation [2]. Unfortunately, a prophylactic vaccine is not currently available, and the standard therapy with a pegylated interferon-α/ribavirin combination has limited efficacy depending on HCV genotypes, and is accompanied with substantial side effects [3]. Therefore, further vigorous investigation of HCV is warranted, particularly into its complete viral life cycle and the virus-host interaction.

A cell culture system for HCV infection became available in 2005 using the HCV genotype 2a strain [4], [5], that was originally isolated from a patient with fulminant hepatitis C [6] and that has a unique capacity for high replication without requiring adaptation to the cell culture system [7]. JFH-1 is used to create cell culture-produced HCV (HCVcc) via transfection of *in vitro*-transcribed JFH-1 RNA into Huh-7 human hepatoma cells; it is infectious for naïve Huh-7 cells *in vitro* and for chimpanzees *in vivo* [4]. Efficiencies of production and infection of HCVcc have been greatly improved by using permissive cell lines such as Huh-7.5 cells, which were derived from HCV RNA replicon-transfected Huh-7 cells by eliminating HCV RNA with type I or type II interferons [5], [8]. The HCVcc system has also been applied for studies of other HCV strains, as chimeric forms based on the JFH-1 strain [8]–[13].

The production and infection of HCVcc *in vitro* each require quantification of infectious HCV virions. For this purpose, a focus-forming assay of HCV virions is typically performed by immunostaining HCV antigens with fluorochrome-conjugated specific antibodies and subsequent fluorescence microscopical observation. However, manual counting of foci by fluorescence microscopical observation is inconvenient and labor-consuming, and can yield biased results. Therefore, in the present study, we established and optimized a method of colorimetric focus-forming assay and image analysis, using an ELISpot reader for convenient and objective quantification of HCV virions.

**Figure 1:**
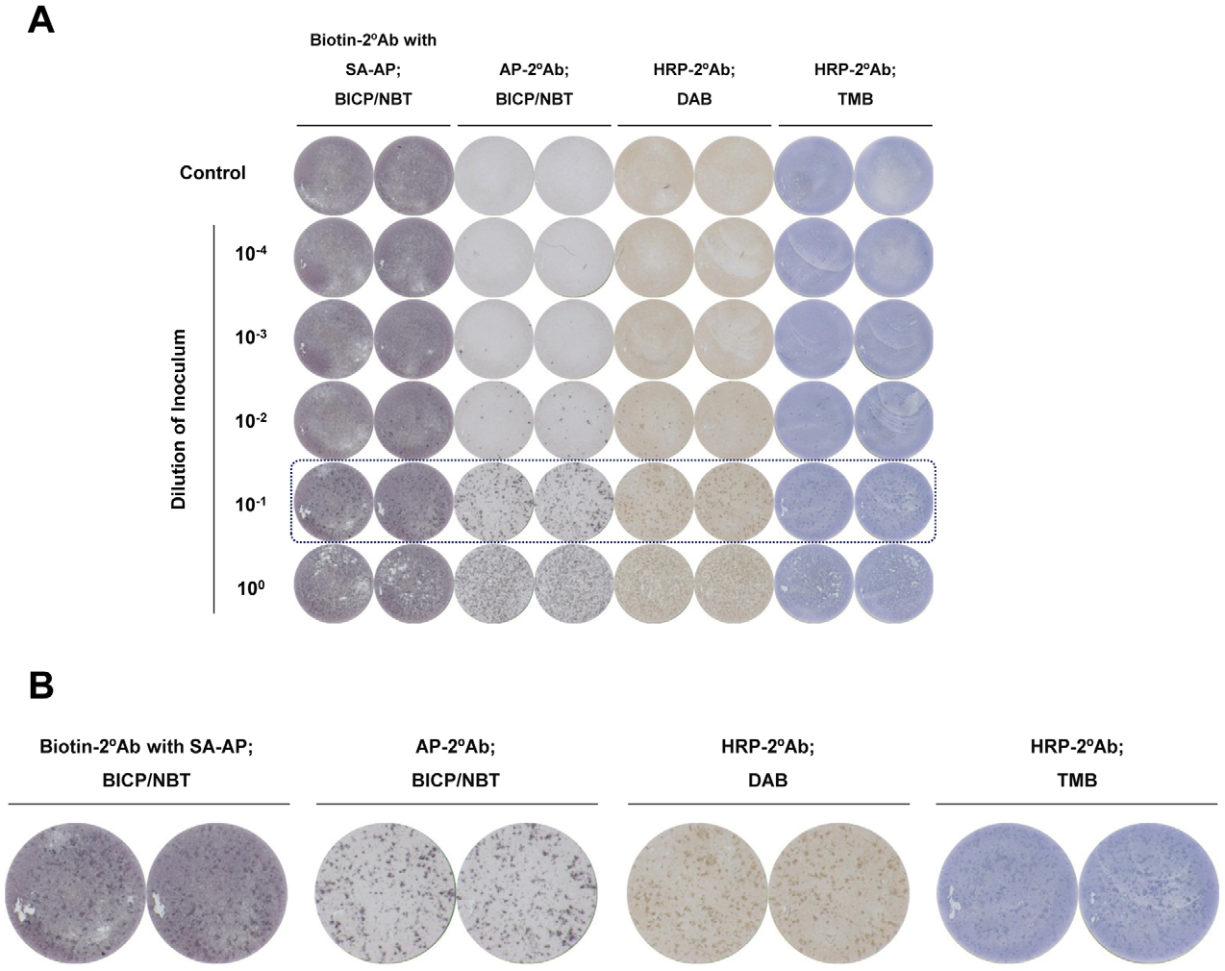
Comparison of colorimetric focus-forming assays using various secondary antibodies and chromogenic substrates. (A) Huh-7.5 cells inoculated with serial dilutions of HCV were immunostained with monoclonal anti-HCV core antibody and secondary antibodies conjugated with different enzymes, as indicated, followed by chromogenic development using various substrates, and image scanning by ELISpot reader. (B) A magnified view of the scanned image of the colorimetric focus-forming assay revealed that alkaline phosphatase-conjugated secondary antibody with BCIP/NBT yielded the best results, considering background and distinctness of the foci. Biotin-2°Ab, biotin-conjugated secondary antibody; SA-AP, streptavidin-conjugated alkaline phosphatase; AP-2°Ab, alkaline phosphatase-conjugated secondary antibody; HRP-2°Ab, horseradish peroxidase-conjugated secondary antibody; BCIP/NBT, 5-bromo-4-chloro-3-indolyl phosphate/nitro blue tetrazolium; DAB, 3,3′-diaminobenzidine; TMB, 3,3′,5,5′-tetramethylbenzidine.

## Results

### Establishment and optimization of colorimetric focus-forming assay for HCV virions

An immunofluorescence-based focus-forming assay is often used for quantification of HCV virions; however, it is a laborious and time-consuming procedure, and can yield biased results. Therefore, we established a colorimetric focus-forming assay for more convenient and objective analysis. A monolayer of Huh-7.5 cells in a 96-well tissue culture plate was infected with various dilutions of JFH-1 HCVcc, followed by chromogenic development; the results were scanned by ELISpot reader for automated focus counting.

**Figure 2:**
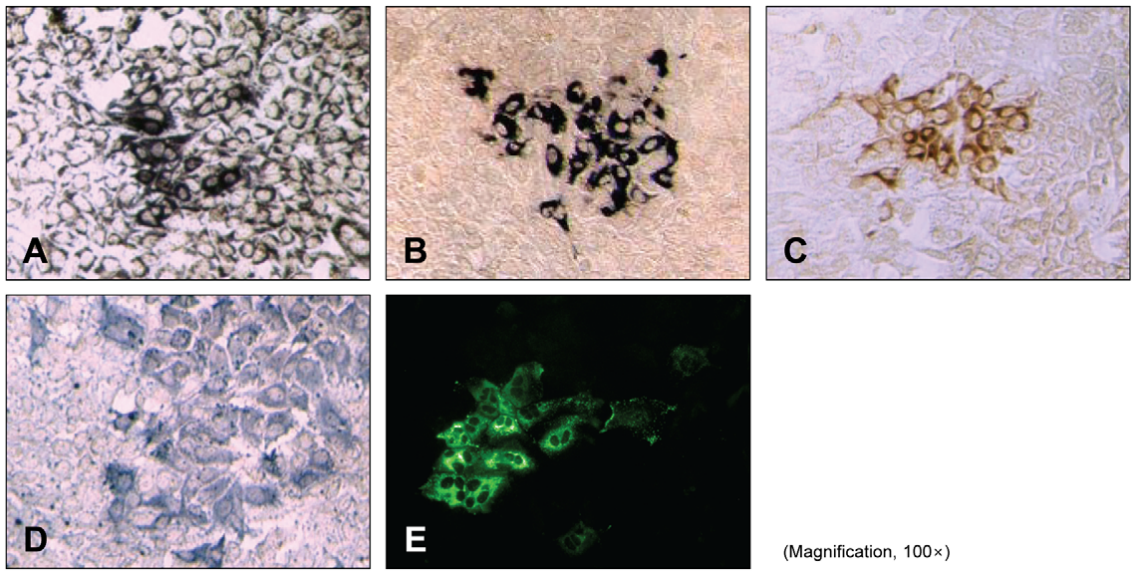
Microscopic images of HCV foci, developed using various secondary antibodies and chromogenic substrates. (A) Biotin-conjugated secondary antibody in conjunction with streptavidin-conjugated alkaline phosphatase and BCIP/NBT. (B) Alkaline phosphatase-conjugated secondary antibody and BCIP/NBT. (C) Horseradish peroxidase-conjugated secondary antibody and DAB. (D) Horseradish peroxidase-conjugated secondary antibody and DAB. (E) Fluorescence-conjugated secondary antibody. Magnification, 100×.

First, we optimized the assay by testing different enzymes with various chromogenic substrates. Use of biotin-conjugated secondary antibody and streptavidin-conjugated alkaline phosphatase with BCIP/NBT chromogenic substrate resulted in background intensity that was too high to discriminate the foci (Figure 1), and the foci were not clear when horseradish peroxidase-conjugated secondary antibody was used with DAB or TMB chromogenic substrate (Figure 1). Microscopic observation demonstrated that alkaline phosphatase-conjugated secondary antibody with BCIP/NBT chromogenic substrate provided the best results, showing clear focus with minimal background (Figure 2), with distinctness comparable to that observed in an immunofluorescence assay. The colored foci were well detected using different anti-HCV primary antibodies, including anti-HCV core (Figure 1 and 2) and anti-HCV NS3 (Figure S1).

**Figure 3:**
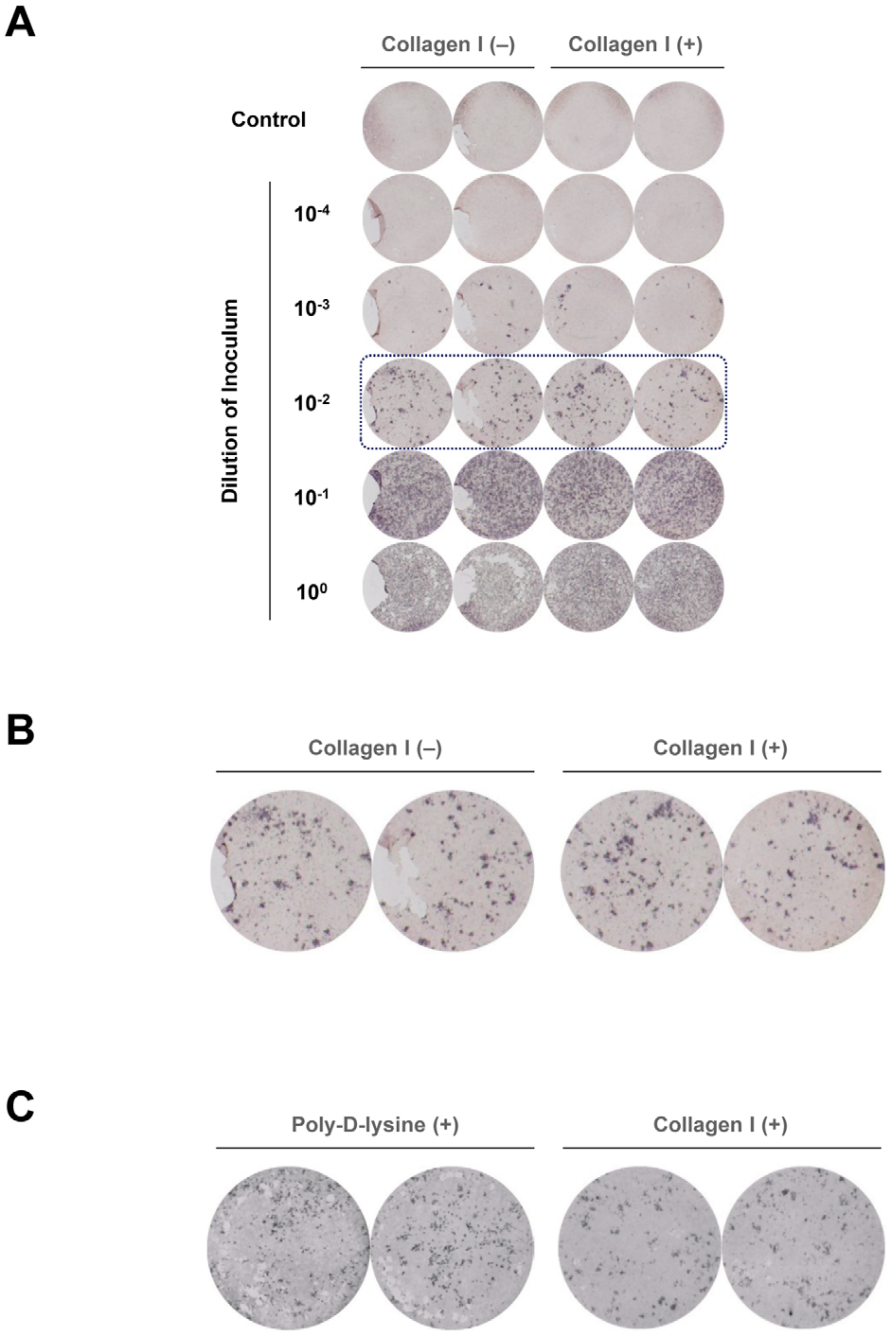
Prevention of cell detachment by type I collagen pre-coating. (A) The scanned image of a colorimetric focus-forming assay emphasizes the importance of pre-coating the plate with type I collagen for preventing cell detachment during the vigorous washing steps in the assay. (B) Magnified view of boxed images in (A). (C) The culture plate was pre-coated with either poly-D-lysine 4 μg/cm^2^ or type I collagen 5 μg/cm^2^, and the colorimetric focus-forming assay was performed. Type I collagen coating was superior to poly-D-lysine coating in terms of prevention of cell detachment.

We tested if the colorimetric focus-forming assay worked for HCV strains other than JFH-1. We mimicked a setting of focus-forming assay by culturing a small number of serially-diluted genotype 1a H77-replicon Huh-7.5 cells with a large number of non-replicon Huh-7.5 cells. The colorimetric assay worked in this setting (Figure S2A), and focus-like regions were identified by image analysis (Figure S2B). In microscopic observation, focus-like regions were well distinguished from surrounding HCV antigen-negative cells (Figure S2C).

**Figure 4:**
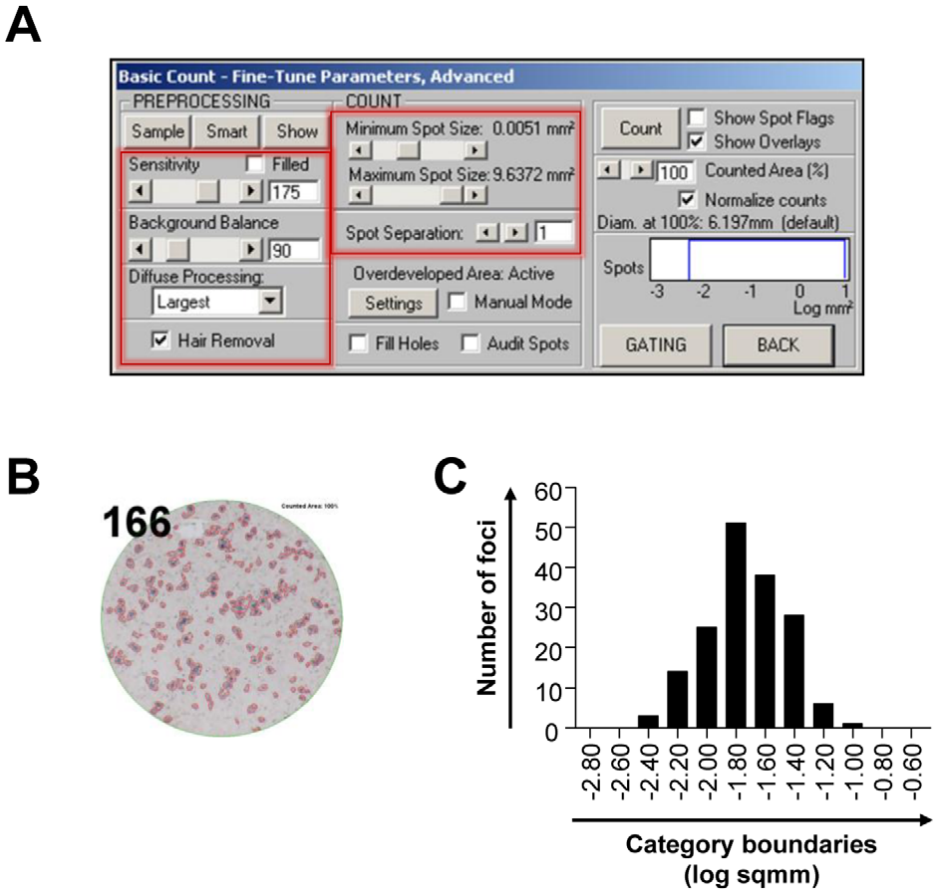
Automated quantification of HCV foci by image analysis. (A) Several parameters, including spot size, sensitivity, background balance, and others, were adjusted to define the foci for automated focus counting. (B) The defined foci are encircled in red, and the number of counted foci is denoted in the left upper corner. (C) A histogram depicting the distribution of foci size in a well.

We also optimized pre-coating reagents for 96-well plates, to minimize detachment of Huh-7.5 cells during the vigorous washing steps. We found that type I collagen was useful for this purpose (Figure 3A and B). Type I collagen coating yielded a better result than poly-D-lysine coating [14] in terms of prevention of cell detachment (Figure 3C). In a poly-D-lysine-coated well, there were many small holes as a result of cell detachment. Therefore, for further analyses, we used alkaline phosphatase-conjugated secondary antibody with BCIP/NBT chromogenic substrate in type I collagen-coated plates.

**Figure 5:**
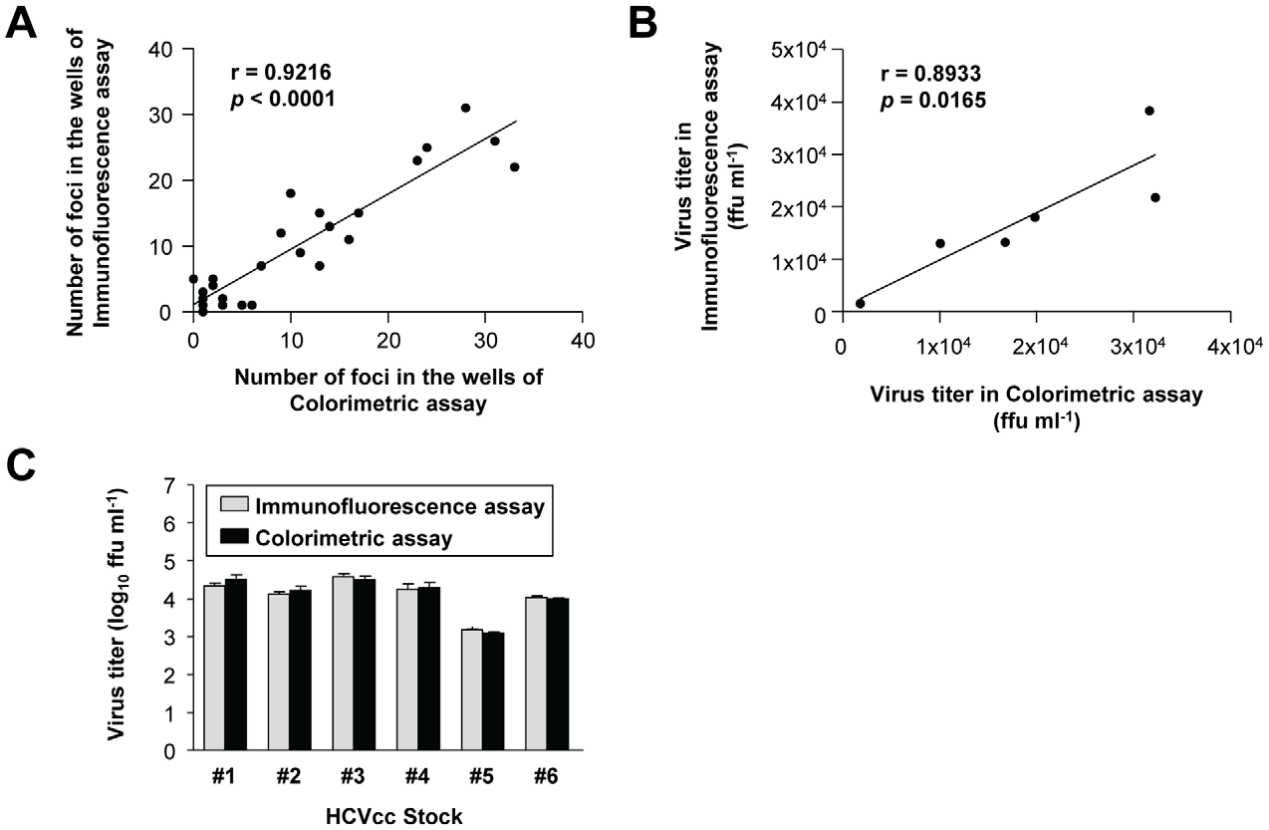
Comparison of colorimetric assay with immunofluorescence-based focus-forming assay. (A) The number of foci in each well determined by colorimetric focus-forming assay with image analysis strongly correlated with that determined by immunofluorescence-based focus-forming assay and manual counting. (B) The virus titers, calculated using the foci numbers obtained by the two different methods, also showed significant correlation. (C) The determined viral titers in six different batches of HCVcc indicated no significant differences between the results of the two types of focus-forming assay.

### Quantification of HCV foci by image analysis

Next, we counted the number of foci using an ELISpot reader and image analysis software. To define the focus for the automated focus-counting function of the software, we adjusted several parameters, including spot size, sensitivity, background balance, and others. An example of the adjusted parameters for foci counting is presented in Figure 4A, and the defined foci are marked in Figure 4B. The distribution of foci size in a well is presented in Figure 4C. The colorimetric focus-forming assay and subsequent image analysis enabled us to easily count the number of foci without laborious manual counting under fluorescence microscope.

### Comparison of colorimetric vs. immunofluorescence-based focus-forming assay

Finally, we compared the foci number determined using the colorimetric focus-forming assay and image analysis with that counted by immunofluorescence-based focus-forming assay. We performed both types of focus-forming assay in six different batches of JFH-1 HCVcc. We found a strong correlation between the foci number determined by image analysis following a colorimetric focus-forming assay and the number determined by immunofluorescence-based focus-forming assay and manual counting (Figure 5A). The foci number was used to determine the virus titer in each batch of HCVcc. The virus titer determined by colorimetric focus-forming assay and image analysis significantly correlated with that determined by immunofluorescence-based focus-forming assay (Figure 5B), with no significant difference between the two types of focus-forming assays (Figure 5C).

## Discussion

The JFH-1-based HCVcc system enables detailed analysis of HCV in viral life cycle, virus-host interaction, and antiviral innate immunity, which facilitates development of anti-HCV drugs. Experiments using HCVcc require consistent experimental conditions; for example, *in vitro* studies of HCVcc infection require a consistent multiplicity-of-infection (MOI) in a series of experiments. Thus, infectious HCV virions must be quantified in each batch of HCVcc. For this purpose, immunofluorescence-based focus-forming assay is performed by immunostaining of HCV antigens with fluorochrome-conjugated specific antibodies, followed by fluorescence microscopical observation. However, manual counting of foci by fluorescence microscopical observation is a cumbersome procedure and can yield biased results. In the present study, therefore, we established and optimized a method for a colorimetric focus-forming assay and automated image analysis using an ELISpot reader, which can replace the conventional immunofluorescence-based focus-forming assay for more convenient and objective quantification of HCV virions.

The image-analyzing function of the ELISpot reader has previously been successfully used for plaque-forming assays of cytopathic viruses [15], [16]. However, a plaque-forming assay cannot be performed for non-cytopathic viruses; instead, the virus-infected foci are typically stained with viral protein-specific antibodies for a focus-forming assay, and the immunostained foci are often visualized by fluorescence. With this method, researchers must count the foci manually under fluorescence microscope and cannot be aided by the image-analyzing function of the ELISpot reader. In contrast, the colorimetric focus-forming assay established in the present study is easily analyzed by the spot-counting function of the ELISpot reader. The application of this assay may not be limited to HCV, but can likely also be extended to other non-cytopathic or minimally cytopathic viruses that are currently quantified by focus-forming assay [17]–[19].

The JFH-1-based HCVcc system was successfully established due to uniquely high replication capacity of JFH-1 strain. HCV-permissive cell lines, such as Huh-7.5, show limited infection *in vitro* by any HCV strain that is not chimerically recombined with the JFH-1 backbone. This limitation of the HCVcc system restricts the usage of the presented colorimetric focus-forming assay and image analysis method. However, more efficient HCVcc systems may be developed in the future, such that a broad range of HCV strains, including clinical isolates, will be able to infect HCV-permissive cell lines *in vitro*. In this event, the colorimetric focus-forming assay and image analysis method that have been established and optimized in the present study will be applicable for additional purposes, including screening of anti-HCV neutralizing antibodies and HCV entry blockers.

## Materials and Methods

### Cell culture

Huh-7.5 cells (provided by Apath, LLC, Brooklyn, NY) were cultured at 37°C with 5% CO_2_ in Dulbecco's Modified Eagle Medium (DMEM; Welgene, Daegu, Korea) supplemented with 10% fetal bovine serum (Welgene), 4.5 g/L glucose, L-glutamine, and 1% penicillin/streptomycin (Invitrogen, Carlsbad, CA).

### Reagents

The antibodies used in this study included mouse monoclonal anti-HCV core IgG_1_ (Clone C7–50; Thermo Scientific/Affinity BioReagents, Rockford, IL), anti-HCV NS3 IgG_1_ (Clone BDI371; Meridian Life Science, Memphis, TN), Alexa Fluor 488-conjugated anti-mouse IgG (H + L) (Invitrogen/Molecular Probes, Eugene, OR), alkaline phosphatase-conjugated rabbit anti-mouse IgG (Sigma-Aldrich, Saint Louis, MO), horseradish peroxidase-conjugated goat anti-mouse Ig (BD Biosciences/Pharmigen, San Diego, CA), biotin-conjugated goat anti-mouse Ig (BD Biosciences/Pharmigen), and streptavidin-conjugated alkaline phosphatase (Invitrogen). The substrates for chromogenic development included the AP Conjugate Substrate Kit (Bio-Rad, Hercules, CA), consisting of 5-bromo-4-chloro-3-indolyl phosphate (BCIP)/nitro blue tetrazolium (NBT) chromogenic substrate, 3,3′-diaminobenzidine (DAB; Vector Laboratories, Burlingame, CA), and 3,3′,5,5′-tetramethylbenzidine (TMB; Sigma-Aldrich). Rat tail collagen type I was obtained from BD Biosciences (Bedford, MA). Poly-D-lysine was obtained from Sigma-Aldrich (Saint Louis, MO).

### HCV cell culture (HCVcc)

The JFH-1 strain (genotype 2a) of HCV was produced by transfecting Huh-7.5 cells with *in vitro* transcribed RNA from a plasmid encoding the full JFH-1 HCV genome (provided by Apath). Huh-7.5 cells were transfected with DMRIE-C reagent (Invitrogen). Following RNA transfection, cell culture supernatants from the peak of HCV production were used to infect naïve Huh-7.5 cells. The HCV-infected Huh-7.5 cells were passaged, and cell culture supernatants with the highest HCV production were selected, as described previously [14]. The selected HCV supernatants, or HCVcc stocks, were filtered (0.45 μm) and frozen at −70°C until use.

### Immunofluorescence-based focus-forming assay

Cells were prepared at 1×10^5^/ml complete DMEM, and seeded at 100 μl/well into transparent 96-well flat-bottom tissue culture plates. After overnight plating at 37°C (5% CO_2_), 50 μl/well of medium was removed from each well, and 100 μl/well of serial ten-fold dilutions of complete medium containing the virus was added; inoculation with each dilution was performed in at least duplicate. After further incubation at 37°C for 72 h, culture medium was completely removed, followed by fixation/permeabilization with 50 μl/well of ice-cold 100% methanol at −20°C for 15 min. The fixed and permeabilized cells were washed twice with 200 μl/well of PBS, followed by blocking in 50 μl/well of blocking buffer (1% BSA, 0.25 mM EDTA in PBS) at room temperature for 1 h. The blocking buffer was then replaced with 40 μl/well of mouse monoclonal anti-HCV core IgG_1_ (1∶300 in blocking buffer) for 1 h at room temperature. After washing three times with 200 μl/well of PBS, the cells were subjected to 40 μl/well of AlexaFluor-488-conjugated anti-mouse IgG secondary antibody (1∶1,000 in blocking buffer) for 1 h at room temperature, protected from light. The cells were washed three times with 200 μl/well of PBS, followed by addition of 50 μl/well of PBS for fluorescence microscopy. The infected cell foci were visualized using a Zeiss inverted microscope (Carl Zeiss, Thornwood, NY). The entire plate was viewed, and the well containing the last dilution in which the infected cell focus was visible was identified. The foci were manually counted only in the last two wells, and the viral titer was calculated from the average number of foci from the last two wells in duplicate. For example, if the number of counted foci were 84 and 70 in the 1∶100 diluted duplicate wells and 7 and 9 in the 1∶1,000 diluted duplicate wells, the original concentration was calculated as follows: [(84+70)×100+(7+9)×1,000]/4/100×1,000 = 7.85×10^4^ virions/ml.

### Colorimetric focus-forming assay

Cells were prepared as described above, in 96-well flat-bottom tissue culture plates that were pre-coated with rat tail collagen type I at 5 μg/cm^2^ or poly-D-lysine at 4 μg/cm^2^. Inoculation with serially diluted infectant, fixation/permeabilization, blocking, and staining with primary antibody were performed as described for the immunofluorescence-based focus-forming assay. For secondary antibodies, we used 40 μl/well of either alkaline phosphatase-conjugated rabbit anti-mouse IgG (1∶200 in blocking buffer), horseradish peroxidase-conjugated goat anti-mouse Ig (1∶1,000 in blocking buffer), or biotin-conjugated goat anti-mouse Ig (1∶400 in blocking buffer) with subsequent use of streptavidin-conjugated alkaline phosphatase (1∶5,000 in blocking buffer). For chromogenic development, we used 100 μl/well of the AP Conjugate Substrate Kit (Bio-Rad) consisting of BCIP/NBT chromogenic substrate for assays with alkaline phosphatase-conjugated secondary antibody, or 100 μl/well of either DAB or TMB chromogenic substrate for assays with horseradish peroxidase-conjugated secondary antibody. Chromogenic development was monitored at room temperature until optimal development, and the reaction was stopped by rinsing twice with 200 μl/well of PBS. Immediately after complete removal of PBS from each well, the plate was scanned by an ELISpot reader (ImmunoSpot® S5 Versa Analyzer, Cellular Technology Ltd., Shaker Heights, OH), and was subjected to automated focus counting.

### Automated focus counting using image analysis

An image of the assay plate was acquired using the ELISpot reader, and automated focus counting was performed using BioSpot 5.0 Professional software (Cellular Technology Ltd.). The focus-counting parameters were adjusted to optimized settings depending on the strength of chromogenic reaction.

### Statistical analysis

Data are presented as the mean ± standard error of the mean (SEM). Pearson's correlation coefficient was used to analyze the correlation between colorimetric focus-forming assay with image analysis and automated foci counting, and immunofluorescence-based focus-forming assay with manual foci counting. Two-tailed Student's *t*-test was performed to determine significant difference in viral titers between colorimetric and immunofluorescence-based focus-forming assays. All statistical analysis was done using GraphPad Prism version 5.0 (GraphPad Software, San Diego, CA). A *p* value of less than 0.05 was considered statistically significant.

## Supporting Information

Figure S1 **Colorimetric focus-forming assay using anti-HCV NS3 primary antibody.** (A) Huh-7.5 cells inoculated with serial dilutions of HCV were immunostained with monoclonal anti-HCV NS3 antibody and alkaline phosphatase-conjugated secondary antibody, followed by chromogenic development using BCIP/NBT. (B) Magnified view of boxed images in (A).
(TIF)

Figure S2 **Colorimetric assay for detection of focus-like regions in genotype 1a H77-replicon Huh-7.5 cells.** (A) A small number of genotype 1a H77-replicon Huh-7.5 cells were serially diluted and cultured with a large number of non-replicon cells. After 5 days of culture, the cells were immunostained with monoclonal anti-HCV core antibody and alkaline phosphatase-conjugated secondary antibody, followed by chromogenic development using BCIP/NBT. (B) The focus-like regions were readily detected by image analysis. (C) A microscopic image of colored focus-like regions formed by genotype 1a H77-replicon Huh-7.5 cells. Magnification, 100×.
(TIF)
