# Supplementary figures and images for: Colorimetric Focus-Forming Assay with Automated Focus Counting by Image Analysis for Quantification of Infectious Hepatitis C Virions
Source: PLoS One. 2012 Aug 24;7(8):e43960. doi: 10.1371/journal.pone.0043960 (PMC3427175; doi:10.1371/journal.pone.0043960)

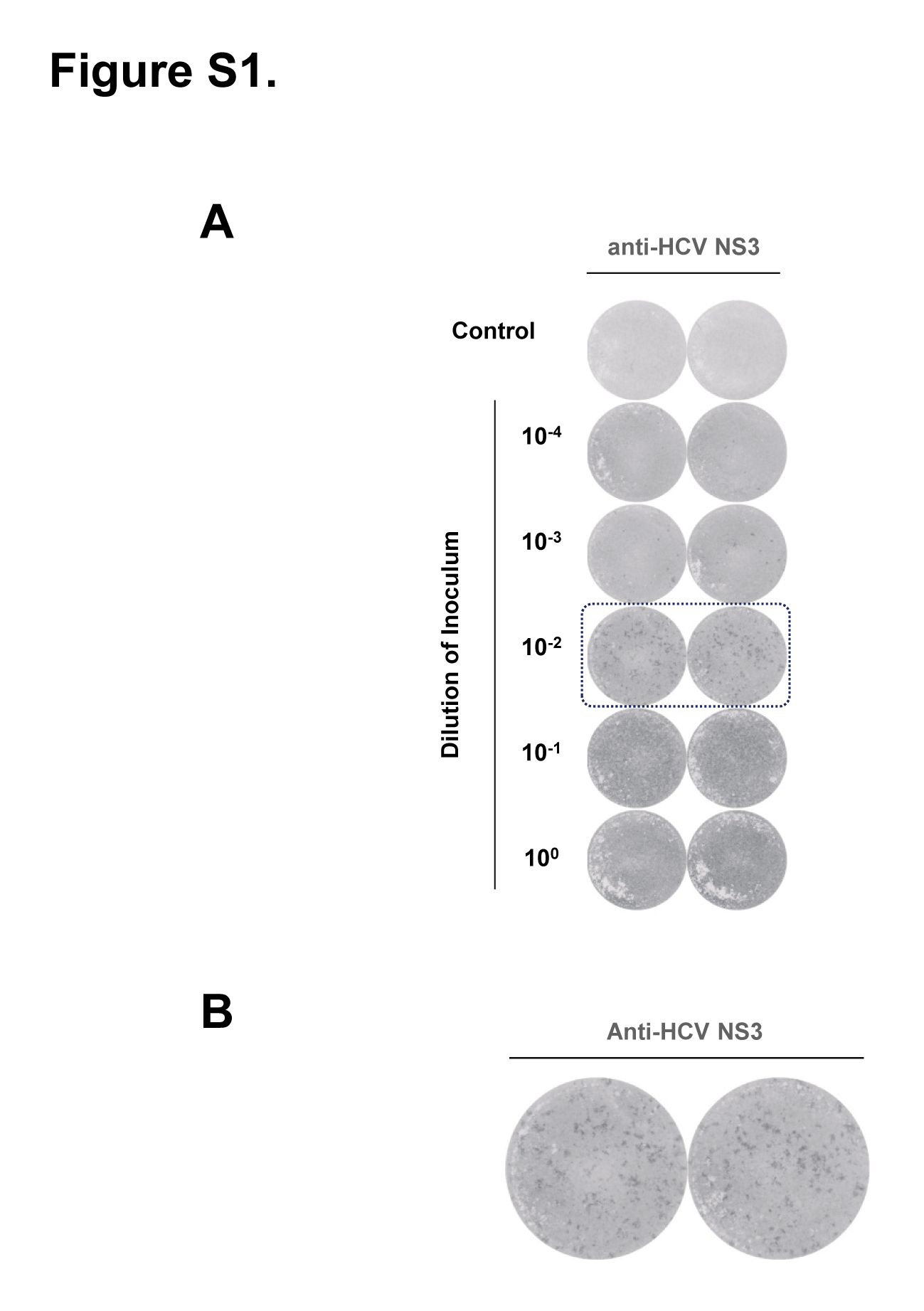

Supplement: Figure S1 — Colorimetric focus-forming assay using anti-HCV NS3 primary antibody. (A) Huh-7.5 cells inoculated with serial dilutions of HCV were immunostained with monoclonal anti-HCV NS3 antibody and alkaline phosphatase-conjugated secondary antibody, followed by chromogenic development using BCIP/NBT. (B) Magnified view of boxed images in (A). (TIF) [file pone.0043960.s001.tif]

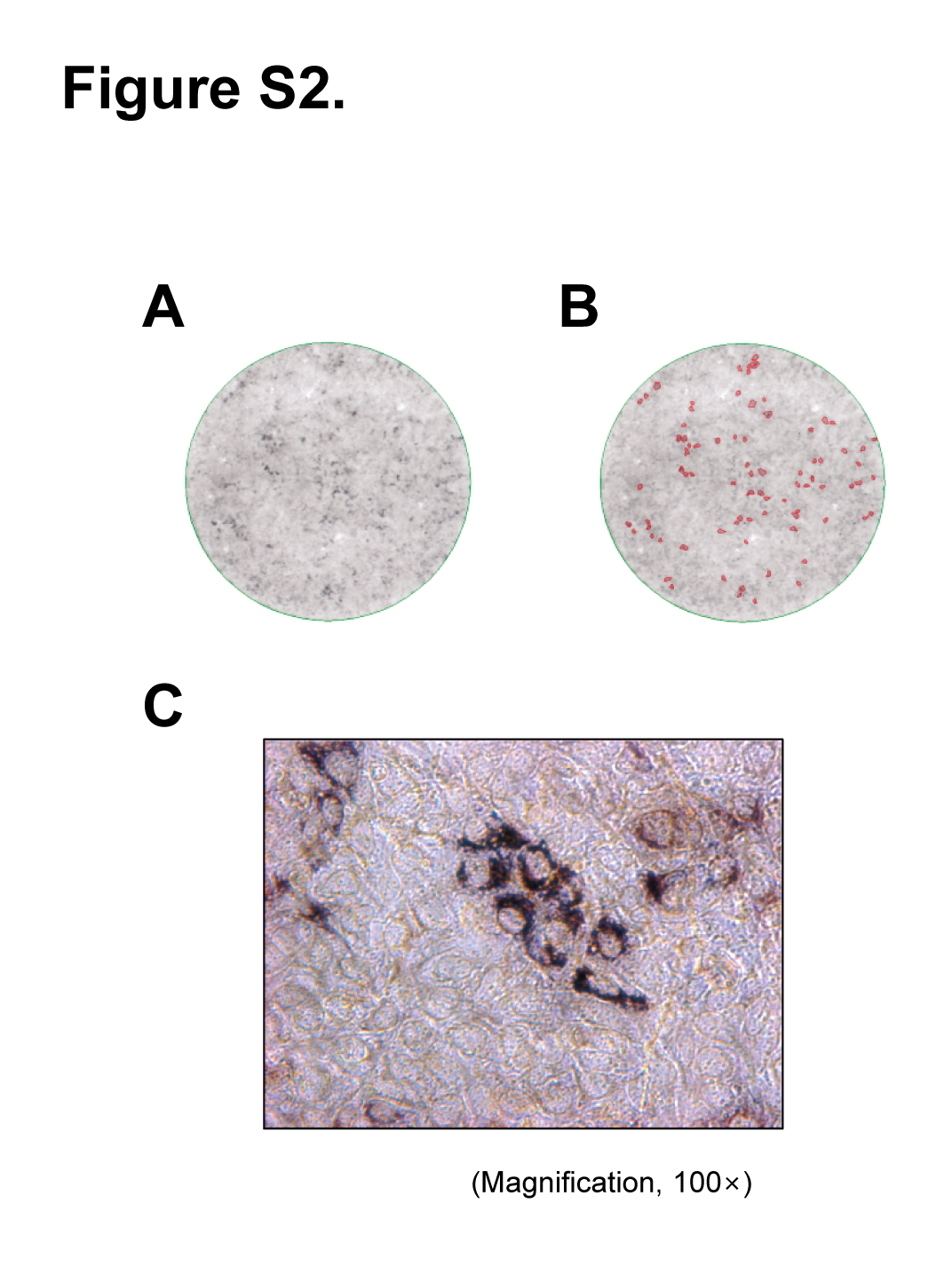

Supplement: Figure S2 — Colorimetric assay for detection of focus-like regions in genotype 1a H77-replicon Huh-7.5 cells. (A) A small number of genotype 1a H77-replicon Huh-7.5 cells were serially diluted and cultured with a large number of non-replicon cells. After 5 days of culture, the cells were immunostained with monoclonal anti-HCV core antibody and alkaline phosphatase-conjugated secondary antibody, followed by chromogenic development using BCIP/NBT. (B) The focus-like regions were readily detected by image analysis. (C) A microscopic image of colored focus-like regions formed by genotype 1a H77-replicon Huh-7.5 cells. Magnification, 100×. (TIF) [file pone.0043960.s002.tif]
